# Protective measures for COVID-19 for healthcare providers and laboratory personnel

**DOI:** 10.3906/sag-2004-132

**Published:** 2020-04-21

**Authors:** Canan AĞALAR, Derya ÖZTÜRK ENGİN

**Affiliations:** 1 Department of Infectious Diseases and Clinical Microbiology University of Health Sciences,Fatih Sultan Mehmet Training and Research Hospital, İstanbul Turkey

**Keywords:** COVID-19, healthcare providers, personal protection equipment

## Abstract

In the COVID-19 pandemic, which affects the whole world, healthcare professionals (HCP) are at high risk of transmission due to their direct contact with patients with COVID-19. Therefore, how to ensure the triage of the patient with acute respiratory symptoms should be determined in advance, the contact distance should be arranged to be at least 2 m, COVID-19 suspect or diagnosed patient should be instructed to wear a surgical mask. During the care of these patients, HCP should wear their personal protective equipment (PPE) in accordance with the procedure and should not neglect hand hygiene. The samples of the patient with known or suspected COVID-19, patient should also be known to be risky in terms of contamination, and a risk assessment should be performed for the procedures to be performed in laboratories. The PPE should be used in accordance with the procedure to be performed. The protection of the HCP, who sacrifice at the risk of life, is possible only by complying with infection control and precautions.

## 1. Intoduction

The novel coronavirus (COVID-19) carries a high risk for society and healthcare providers (HCP) because it can be transmitted even when the disease progresses asymptomatically in some patients [1]. The main sources of infection are infected people. The virus is transmitted through droplets and close contact. It is believed that infectivity starts before the symptoms and it significantly decreases 7 days after the onset of symptoms. It is reported that the infectivity period depends on the severity and the stage of the infection of the patient. This virus can survive on nonliving surfaces in 22–25 °C and 40–50% relative humidity for up to 5 days and this increases the risk of infection. Aerosol transmission is also possible [2]. In a study, it has been shown that the virus can remain alive in the aerosol for up to 3 h after aerosol generating procedures and could be detected [3]. 

## 2. Historical background

The usage of personal protective equipment (PPE) reduces the risk of transmission but does not fully eliminate it. In China, it has been reported that 2055 HCP working in 476 different hospitals, mainly from Hubei (88%), have been infected with COVID-19 from December 18, 2019 to February 20, 2020. It is emphasized that the reason for this high rate of infection among HCP stems from shifts that last more than 10 h a day due to the large number of patients and serious staff shortages. Furthermore, excessive fatigue and stress weaken the immune system and therefore, increase the sensitivity to COVID-19. As the infection widened rapidly, it is reported that there has been a rapid decrease in the availability of PPE, and a rapid increase in infection in HCP that resulted in a higher rate of transmission among visitors, personnel, and patients [4]. 

 In a study with 138 patients from China, it was demonstrated that 57 patients (41.3%) were infected in the hospitals, among these patients 17 (12.3%) of them were in the hospital for other reasons and 40 (29%) of them were HCP [4]. Among these infected HCP, 31 (77.5%) were working in clinical services, 7 (17.5%) in emergency service, and 2 (5%) in intensive care units (ICU), respectively [5]. 

## 3. Measures to reduce exposure risk

In order to prevent the transmission from patient to healthcare workers, the necessary precautions should be taken that comprise the whole process, which start with the admission of the patient to the hospital [6]. Further, procedures like elective surgeries and routine check-ups should be postponed. For acute respiratory infection symptoms (ARIS) admissions triage protocols should be created [6,7]. These protocols start with restriction of entry points to the hospitals. The patients who come to the hospitals should be given face masks and be told to wear them at all times. HCP should be fully equipped with PPE and should be ready. The patients who are suspected of COVID-19 should be isolated safely and rapidly [6].

Hospital entrances, patient rooms, and waiting rooms should be equipped with hand disinfectants that are 60–95% alcohol and waste containers that can be used without contact. A physical barrier made of glass or plastic should be placed in order to separate triage personnel and possible infectious patients to restrict close contact. Examination rooms should be big enough so that there can be 2 m between the HCP and the patient. These rooms should also be available for ventilation [6].

HCP that has been in close contact with or work in care of a COVID-19 patient is under risk of getting infected by COVID-19. Centres for Disease Control and Prevention (CDC) defines close contact as, being in the same environment with an infected person without maintaining the 2 m minimum distance and being in direct contact with secretions of the infected person [6]. The national interim guideline of Ireland defines close contact as being in face to face contact with a diagnosed COVID-19 patient without maintaining 2 m distance for more than 15 min. The same guideline also defines performing any procedure that produces aerosol without necessary PPE or being in this room while this procedure is taking place without the necessary PPE, or any contact with the patient, bodily fluids of the patient or laboratory samples of the patient without using necessary PPE as close contact [8]. 

Transmission from HCP to HCP is as important of a transmission way as from patient. One of the measures that will reduce the risk of transmission among HCP is creating teams among providers who work in hospitals and labs. By doing this, social distancing can be maintained, and the risk of cross-infection can be reduced. All HCP should be checked twice a day for ARIS symptoms, and body temperature to increase the chances of early diagnosis. If a member of the team is infected with COVID-19, all close contacts should take quarantine measures [9]. 

### 3.1. Isolation measures for preventing transmission of COVID-19

HCP should take protective measures assuming that everyone is potentially infected or is colonized with a pathogen that can be transmitted in a healthcare environment [6]. In order to prevent COVID-19 transmission HCP should take additional measures for contact during aerosol generating procedures (AGPs) alongside standard measures for droplets, close contact, and airborne transmission [10]. One of the primary measures that will reduce the transmission in health institutions is ensuring hand hygiene [1]. 

#### 3.1.1. Hand hygiene

HCP should ensure their hand hygiene before and after contact with a patient, after contact with potentially infected material, and before and after using PPE. Since bare hands can get contaminated while taking off PPE, performing necessary steps to ensure hand hygiene is of great importance. HCP should wash their hands or at least 20 s using soap and water or should disinfect their hands with 60–95% alcohol-based hand disinfectant. If the hands got visibly contaminated, they should be washed with soap and water [6,10]. The hand hygiene should also be ensured before and after going into ICUs [11]. 

#### 3.1.2. Personal protective equipment 

Ineffectiveness of PPE may contribute to nosocomial transmission of COVID-19 [12]. HCP should be educated on when to use which PPE, how to put on, take off, and change them by themselves to prevent contamination and on how to properly discard and disinfect this equipment. Health institutions should have procedures and policies that describe the correct order of donning and doffing these PPE in a safe manner [6]. The order for donning the PPE after performing hand hygiene is gown, mask, goggles, face shield, and gloves; the order for doffing the PPE is gloves, face shield, goggles, gown and mask. The mask should be kept until the HCP leaves the contaminated area. The mask should be properly taken off once the contaminated area is left, furthermore it is important not to neglect hand hygiene once all of these items are removed [11]. The PPE that should be used for HCP according to the procedures have been defined by the World Health Organization (WHO) [10]. Recommendations about using of PPE are given in Table [13–16].

**Table 1:** Recommended type of personal protective equipment (PPE) in the context of COVID-19 disease [13–16].

Setting	Target staff	Activity	Type of PPE
COVID-19 patient room in clinics	Healthcare workers	Providing direct care	Surgical mask GlovesGownGoggles/face shield
		Aerosol-generatingprocedures performed on COVID-19 patients	FFP2 mask Gloves Long-sleeved water-resistant gown Goggles and face shield
	Cleaners	Entering the room of COVID-19 patients	FFP2 mask Gloves Gown Goggles or face shieldBoots or closed work shoes
Ambulance or transfer vehicle COVID-19 patient	Healthcare workers	Transporting suspected COVID-19 patients	FFP2 mask Double nonsterile gloves Long-sleeved water-resistant gown Goggles or face shield
Outpatient facilities	Healthcare workers	Patient with respiratory symptom	Surgical mask Gloves Gown Goggles
	Cleaners	After and betweenconsultations of patients with respiratory symptoms	Surgical mask GlovesGown Boots or closed work shoes
Waiting room	Patient		Patients with respiratory symptoms must wear a medical mask. If possible isolate patients with respiratory symptoms, otherwise keep a distance of 1 m from each other.
Laboratory	Laboratory personnel	Working with respiratory samples	FFP2 mask Double nonsterile gloves Long-sleeved water-resistant gown Goggles or face shield

#### 3.1.3. Masks

European Centre of Diseases and Prevention Control (ECDC) states that if there is a shortage of FFP2/FFP3, if the HCP will be in contact with a diagnosed or suspected COVID-19 case, if there is no risk of aerosol transmission, surgical masks (alongside eye protection, gown, and gloves) can be used. However, ECDC states that if the HCP will be performing procedures like sample collecting that will generate aerosol, they should use FFP2/FFP3 masks that provide high-level protection [7]. Unless indicated by the producer, if the mask that is being used for sample collection has not been damaged, moistened, and/or soiled, it can be used for contacting multiple patients for a maximum of 4–6 h [7].

According to the COVID-19 handbook prepared by the Chinese colleagues, all HCP in all health institutions should wear a medical mask. All staff working in the emergency department, the outpatient department of respiratory care, the outpatient department of infectious diseases, department of stomatology, or endoscopic examination room should wear a medical protective mask such as an N95 mask [17]. 

 The mask should be placed on your face carefully, and there should be no gap between the face and the mask [13]. It is reported that facial hair such as the beard could prevent the mask from sitting and can lessen the protective effect [10,11].

#### 3.1.4. Eye protection

The transmission through the eye is not certain for COVID-19 but as proven with animal experiments the transmission in this way is possible. Therefore, eye protection should not be neglected and should be considered as a part of PPE [18]. HCP should wear eye protection or a disposable face shield that covers the front and sides of the face when going into a patient’s room. Personal eye glasses or contact lenses do not protect the eye sufficiently from the transmission. The eye protection should be taken off before leaving the patient room or care areas. Reusable eye protectors should be cleaned and disinfected before reuse according to the producers’ instructions [6]. 

#### 3.1.5. Gloves

When entering the patient rooms or care areas HCP should wear clean unsterile gloves. If the gloves are ruptured or contaminated hand hygiene should be ensured, and gloves should be changed with new ones. When leaving the patient rooms or care areas the gloves should be removed and hand hygiene should be ensured [6]. Gloves should not be washed and reused [19]. 

#### 3.1.6. Disposable aprons and gowns

Before entering the patients’ rooms or care areas HCP should wear a clean isolation gown and should change it when it gets contaminated. Before leaving these areas, HCP should remove the gown and should dispose it accordingly (red waste container). Reusable gowns should be washed after each use. If there is a shortage of gown, the priority should be given to AGPs, procedures that have the risk of spatter and have a high risk of contamination of HCP due to the possibility of pathogens’ contact with HCP’s clothes and hands [6]. 

The guideline from the United Kingdom recommends the usage of disposable plastic aprons to protect the contamination during patient care. Long sleeved disposable fluid repellent gowns must be worn during AGPs [20].

During the pandemic, one of the most important problems is the availability of PPE. In order to minimize exposure of HCP and minimize the usage of PPE, a group of personnel can be designated to work in the care of these patients [6]. HCP should not touch eye protectors and masks. When the eye protector and masks got damaged, got contaminated and when the HCP leaves the unit they should be changed, and hand hygiene should be ensured [6]. 

Medical equipment that will be used for patients should be specific to patients and they should not be taken out of the rooms or should not be used for other patients. If the equipment like stethoscope and thermometers are being used for more than one patient they should be cleaned and disinfected after every use with, for example, ethyl alcohol (70%). Two medical waste containers should be available, 1 inside and 1 outside of the patient rooms so that the used PPE can be properly discarded [16]. 

## 4. Aerosol generating procedures 

Aerosol generating procedures are intubation, extubation and related procedures, such as manual ventilation and open suctioning of the respiratory tract, tracheotomy/tracheostomy procedures, bronchoscopy, surgery and postmortem procedures involving high-speed devices, noninvasive ventilation (NIV), e.g., bi-level positive airway pressure (BiPAP) and continuous positive airway pressure ventilation (CPAP), induction of sputum, some dental procedures (e.g., high-speed drilling), high-flow nasal oxygen (HFNO), high-frequency oscillating ventilation (HFOV), cardiopulmonary resuscitation [20,21]. Collection of diagnostic respiratory specimens for COVID-19 is considered as an AGP since it can induce coughing reflex [7]. 

### 4.1. Precautions for aerosol generating procedures 

 During AGPs, HCP should use long-sleeved disposable fluid repellent gown (covering the arms and body), higher protection masks such as N95/FFP3 mask, full-face shield or visor and gloves on suspected and confirmed cases [6,20]. Fit test should be done before starting the procedure [22,23]. Only the necessary personnel should perform the procedures and should be allowed in the area of the procedure. These procedures should be performed in respiratory tract isolation rooms, the surfaces in these rooms should be cleaned and rooms should be disinfected after every procedure [6]. 

After AGPs the surrounding can get heavily contaminated. In an enclosed area the time for the aerosol to be cleaned depends on the presence of mechanic/natural ventilation. The time for the sufficient cleaning of aerosols (the time that has to pass until an HCP can get in the room without using FFP3 masks) depends on the air change per hour (ACH) in the room. It is recommended that general wards and single rooms have a minimum of 6 ACH while negative pressure isolation rooms should have a minimum of 12 ACH. The higher the ACH, the faster the aerosol cleaning will be. It is believed that first air change reduces the contaminants in the air by 63% and after 5 times only less than 1% of the contaminants stay in the air [14].

After the patient is discharged or transferred to another room, it is recommended to leave the room empty for 20 min before cleaning if this is a negative pressure isolation room. If this is a neutral pressure room, the windows should be opened and the room should be left that way for an hour before cleaning [14]. 

New and Emerging Respiratory Virus Threats Advisory Group (NERVTAG) reports that the aerosol produced during nebulization is derived from the fluid in the nebulizer and does not contain viral particles that come from the patient. According to the guideline from the United Kingdom; administration of pressurized-humidified oxygen or medication via nebulization are not considered a significant infectious risk for aerosol generation [20]. However, nebulizer therapy is considered as an aerosol generating procedure by both WHO and CDC [15,24]. 

## 5. Recommendations for environmental cleaning 

Significant environmental contamination by patients causes nosocomial transmission of virus [25]. It has been reported that cleaning environmental surfaces and patient care equipment with water and detergent and applying disinfectants at the commonly used hospital level are sufficient and effective [26]. COVID-19 is sensitive to sodium hypochlorite ( 0.1%–0.5%), 70% ethyl alcohol, povidone-iodine (1% iodine), chloroxylenol (0.24%), 50% isopropanol, 0.05% benzalkonium chloride, %1 cresol soap, or hydrogen peroxide (0.5%–7.0%) (27). All surfaces including standard floor, walls, and objects in COVID-19 isolation areas should be disinfected with solutions that have 1000 mg/L chlorine. Disinfection should be performed 3 times a day and should be repeated each time there is contamination [17].

HCP that is responsible for the cleaning of the environment and disposal should wear appropriate PPE [7].

## 6. COVID-19 laboratory biosafety 

Although it is recommended that all laboratory samples to be considered potentially infectious, analysis of particularly unidentified COVID-19 samples may pose a risk of transmission for laboratory workers [9,28]. All personnel should be educated about the usage of biological agents and the risks that come with them. Each lab should do a risk assessment before performing the planned tests. Appropriate PPE should be determined after a detailed risk assessment and should be used by lab personnel. PPE should consist of gown, gloves, eye protection, shield, and a mask that will be chosen in accordance with the risk posed by the type of procedure [29]. 

Although most laboratory analyses are carried out with automation systems, manual touchpoints that exist in the system increase the risk of transmission and contamination. All personnel working in the laboratory should be informed about the risks that may occur. During the analysis phase, procedures should be determined to minimize the formation of aerosols and droplets [9]. In order to reduce the risk of contamination, patient sample transport should not be carried with pneumatic tubing, and before sending the sample, the lab should be informed [30,31]. 

When personnel is working on blood sampling including for serological tests, they should follow applications and procedures which constitute the basis of Good Microbiological Practices and Procedures (GMPP)[29].

The highlights to be followed in relation to COVID-19 laboratory biosafety are specified by WHO. According to WHO; all procedures should be performed in accordance with risk assessment by personnel who can abide by the necessary protocols. Before the deactivation of any samples, the first procedure should be performed in an approved biosafety cabin (BSC) or a primary containment device. Suspected or diagnosed COVID-19 patients’ samples should be carried as UN3373-“Biological Substance Category B” and the viral cultures and isolates should be carried as Category A UN2814, “infectious substance, affecting human”. Diagnostic procedures like sequencing or NAAT that do not pose the risk of transmission should be performed in bio-safety level 2 (BSL-2) labs. Procedures like virus culture, isolation of the virus or neutralization experiments that carry the risk of transmission should be performed in BSL-3 labs that have air inflow. Suitable disinfectants with proven efficacy against COVID-19 should be used [29]. 

For general surface disinfection of labs, sodium hypo chloride 1000 ppm (0.1%) that has proven effective for viruses that have viral envelopes and specifically for spilled blood 10,000 ppm (%1), 62–71% ethanol, 0.5% hydrogen peroxide, quaternary ammonium compounds, and phenolic compounds can be effective for COVID-19. Not only the type of the disinfectant that is used but also the contact duration of the disinfectant to the surface (for example 10 min), the concentration of the active compound, and the expiration date after the preparation date of the solution is of great importance and should be given great care [29].

The necessities of biosafety measures may induce stress to the laboratory personnel. However, it is possible to reduce the risk of transmission and develop a safe working environment in the laboratory by implementing these measures [9].

In conclusion, HCP experience other hardships like physical and mental exhaustion, stress that comes with triage decisions, and grief of losing their patients and colleagues alongside the risk of infection. It should never be forgotten that HCP is the most important source for public health [32]. Prevention of the widening of the pandemic is only possible with healthy and effective HCP teams. The fact that the transmission speed being very high indicates that it is very urgent and necessary to protect the HCP. For this reason, it is very important that health authorities take a series of urgent measures like giving importance to the safety of the HCP, guidance on how to use PPE properly, increased support in terms of logistics and providing medical equipment, and application of developed disinfection techniques for the hotels that the HCP will reside in throughout the pandemic [4].

## Acknowledgments

The review has not been published anywhere or has not currently being assessed for publication by any journal. All the authors contributed sufficiently in the work to take responsibility for appropriate portions of the content. It has not been received any financial support for this review. English redaction of this article was performed by Ece Ağalar. Canan Ağalar is a member of COVID-19 Advisory Committee of Ministry of Health of Turkey.
